# Genetic Manipulation of Human Intestinal Enteroids Demonstrates the Necessity of a Functional Fucosyltransferase 2 Gene for Secretor-Dependent Human Norovirus Infection

**DOI:** 10.1128/mBio.00251-20

**Published:** 2020-03-17

**Authors:** Kei Haga, Khalil Ettayebi, Victoria R. Tenge, Umesh C. Karandikar, Miranda A. Lewis, Shih-Ching Lin, Frederick H. Neill, B. Vijayalakshmi Ayyar, Xi-Lei Zeng, Göran Larson, Sasirekha Ramani, Robert L. Atmar, Mary K. Estes

**Affiliations:** a Department of Molecular Virology and Microbiology, Baylor College of Medicine, Houston, Texas, USA; b Department of Laboratory Medicine, Sahlgrenska Academy, University of Gothenburg, Gothenburg, Sweden; c Department of Medicine, Baylor College of Medicine, Houston, Texas, USA; Johns Hopkins Bloomberg School of Public Health

**Keywords:** fucosyltransferase 2, glycobiology, histo-blood group antigens, isogenic enteroids, isogenic organoids, noroviruses, secretor status

## Abstract

Human noroviruses (HuNoVs) are the leading cause of nonbacterial gastroenteritis worldwide. Histo-blood group antigen (HBGA) expression is an important susceptibility factor for HuNoV infection based on controlled human infection models and epidemiologic studies that show an association of secretor status with infection caused by several genotypes. The fucosyltransferase 2 gene (*FUT2*) affects HBGA expression in intestinal epithelial cells; secretors express a functional FUT2 enzyme, while nonsecretors lack this enzyme and are highly resistant to infection and gastroenteritis caused by many HuNoV strains. These epidemiologic associations are confirmed by infections in stem cell-derived human intestinal enteroid (HIE) cultures. GII.4 HuNoV does not replicate in HIE cultures derived from nonsecretor individuals, while HIEs from secretors are permissive to infection. However, whether FUT2 expression alone is critical for infection remains unproven, since routinely used secretor-positive transformed cell lines are resistant to HuNoV replication. To evaluate the role of FUT2 in HuNoV replication, we used CRISPR or overexpression to genetically manipulate *FUT2* gene function to produce isogenic HIE lines with or without FUT2 expression. We show that FUT2 expression alone affects both HuNoV binding to the HIE cell surface and susceptibility to HuNoV infection. These findings indicate that initial binding to a molecule(s) glycosylated by FUT2 is critical for HuNoV infection and that the HuNoV receptor is present in nonsecretor HIEs. In addition to HuNoV studies, these isogenic HIE lines will be useful tools to study other enteric microbes where infection and/or disease outcome is associated with secretor status.

## Abstract (precis)

Several studies have demonstrated that secretor status is associated with susceptibility to human norovirus (HuNoV) infection; however, previous reports found that FUT2 expression is not sufficient to allow infection with HuNoV in a variety of continuous laboratory cell lines. Which cellular factor(s) regulates susceptibility to HuNoV infection remains unknown. We used genetic manipulation of HIE cultures to show that secretor status determined by *FUT2* gene expression is necessary and sufficient to support HuNoV replication based on analyses of isogenic lines that lack or express FUT2. Fucosylation of HBGAs is critical for initial binding and for modification of another putative receptor(s) in HIEs needed for virus uptake or uncoating and necessary for successful infection by GI.1 and several GII HuNoV strains.

## INTRODUCTION

Human noroviruses (HuNoVs) are a leading cause of nonbacterial gastroenteritis worldwide. From the first recognition in 1968 that a virus caused an outbreak of HuNoV gastroenteritis in an elementary school in Norwalk, Ohio (1), until 2016, there was no *in vitro* culture system of HuNoV in intestinal epithelial cells. A novel HuNoV culture system using human intestinal enteroids (HIEs) generated from stem cells isolated from human small intestinal crypts is now available and is being used worldwide to study virus replication, inactivation, and neutralizing antibodies (2–9).

Secretor status is highly associated with infection and disease caused by many HuNoV strains based upon findings from human experimental infection studies and evaluation of acute gastroenteritis outbreaks (10–14). Fucosyltransferase 2 (FUT2) is an enzyme expressed in human epithelial cells that catalyzes α1,2-fucosylation of the terminal galactose, preferentially on glycan type 1 chain precursors. Glycosyltransferases coded for by *FUT2* along with *FUT3* and *ABO* genes determine the histo-blood group antigens (HBGAs) found on the epithelial cell surface (15, 16). Persons who lack functional *FUT2* alleles do not express ABH HBGAs on epithelial cells, are designated nonsecretors, and are highly resistant to gastroenteritis caused by some HuNoV strains such as GII.4 viruses. Similarly, HIEs derived from nonsecretor individuals are not susceptible to GII.4 HuNoV infection (2). HIEs and gastrointestinal epithelial cells of secretor-positive individuals also bind norovirus virus-like particles (VLPs) (17, 21). However, while HIEs from secretors are permissive to HuNoV replication, FUT2 expression in conventional cancer-derived cell lines is not sufficient to make cells susceptible to HuNoV replication, suggesting that HBGAs function primarily as an initial attachment factor (18, 19). These data also lead to questions on whether there are additional genetically determined differences in HuNoV susceptibility in addition to secretor status. In this study, we evaluated the direct association between FUT2 function and HuNoV infectivity using HIEs with or without FUT2 expression in the same genetic background.

## RESULTS

### Generation and characterization of isogenic HIE lines.

We previously showed that GII.4 viruses can replicate in HIEs derived from secretor-positive individuals and a GII.3 strain can replicate in both secretor-positive and some secretor-negative lines, recapitulating observations seen in epidemiologic studies (2). We determined the *FUT2* (*Se*) and *FUT3* (*Le*) genotypes of jejunal HIE lines and selected two lines (J2 and J4); both lines express FUT3, eliminating Lewis status as a variable in these studies (Table 1). The J2 cell line is heterozygous wild type (*Se*/*se^428^* [J2*Fut2^+/−^*]); because the *Se* gene is autosomal dominant, the cells are secretor positive. The J4 line has a homozygous *se^428^*/*se^428^* (J4*Fut2^−/−^*) recessive mutation and is secretor negative (20). No other mutations associated with loss of or decreased FUT2 enzymatic function were observed in the J2 or J4 line. To better understand the importance of FUT2 in HuNoV infection, we generated isogenic knockout (KO) and knock-in (KI) HIE lines. A lentivirus-delivered CRISPR/Cas9 construct with a guide RNA targeting the *FUT2* coding region was used to knock out *FUT2* from J2 (J2*Fut2^−/−^*). Single cell selection under puromycin pressure was used to isolate a clonal population, and deletions of the gene in both alleles were confirmed by sequencing. To generate the KI *FUT2* line, we transduced a functional *FUT2* coding sequence driven by a cytomegalovirus (CMV) promoter in a lentivirus into J4 (J4*Fut2^−/−/FUT2^*) cells.

**TABLE 1:** Genotyping and phenotyping of HIE lines used in this study

HIE line	HIE line modification^*a*^	Genotyping results^*b*^			Phenotyping results	
		*FUT2* (secretor gene)	*FUT3* (Lewis gene)	*ABO*	Secretor status	HBGA
J2*Fut2*^+/−^	Not modified	*Se*, *se^428^*	*Le*, *Le*	*OB*	Positive	B, Le^b^
J2*Fut2*^−/−^	CRISPR-Cas9 deletion	*se*^Δ^, *se*^Δ^	*Le*, *Le*	*OB*	Negative	Le^a^
J4*Fut2*^−/−^	Not modified	*se^428^*, *se^428^*	*Le*, *le^202^*^,^*^314^*	*OO*	Negative	Le^a^
J4*Fut2*^−/−/^*^FUT2^*	LV Td CMV expression	*se^428^*, *se^428^*, Se^CMV^	*Le*, *le^202^*^,^*^314^*	*OO*	Positive	Le^b^

a LV Td, lentivirus transduced.

b Se, secretor; Le, Lewis; Δ, deletion. Se^CMV^ indicates that the Se gene was expressed under a CMV promoter. Specific mutations in secretor and Lewis genes are indicated by the superscripts.

**FIG 1:**
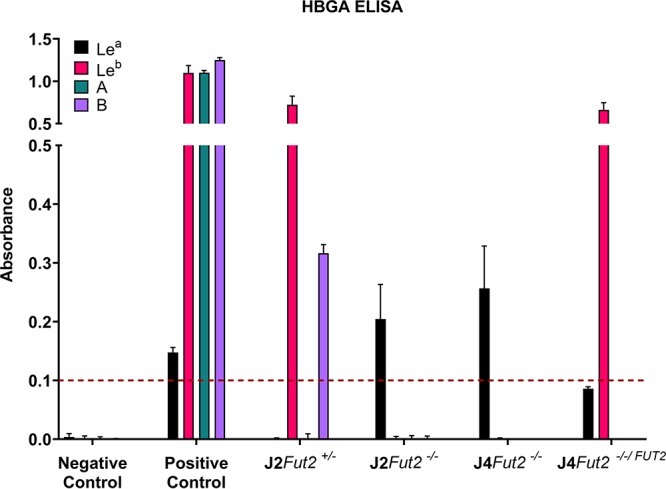
HBGA expression phenotype by ELISA. Five-day differentiated HIEs were evaluated for HBGA expression with primary antibodies against either the Le^a^, Le^b^, A, or B epitope and HRP-conjugated secondary antibodies. Mean absorbance values from two ELISA replicates are plotted. Error bars denote standard deviations (SD). The threshold of detection is indicated by a dashed line at absorbance of 0.1.

First, we confirmed the phenotype of the *FUT2* KO and KI lines by evaluating HBGA expression using an enzyme-linked immunosorbent assay (ELISA) (Fig. 1). J2*Fut2^+/−^* cells expressed the secretor-positive glycans, Le^b^ and B, as expected for this secretor-positive, Lewis-positive *OB* HIE line. KO of *FUT2* altered the J2 phenotype such that the Le^b^ and B glycans were no longer present, and only Le^a^ was detected. J4*Fut2^−/−^* cells expressed Le^a^ but not Le^b^ or other secretor glycans, confirming the secretor-negative genotype of this line. KI of *FUT2* in J4 cells led to Le^b^ instead of Le^a^ expression.

**FIG 2:**
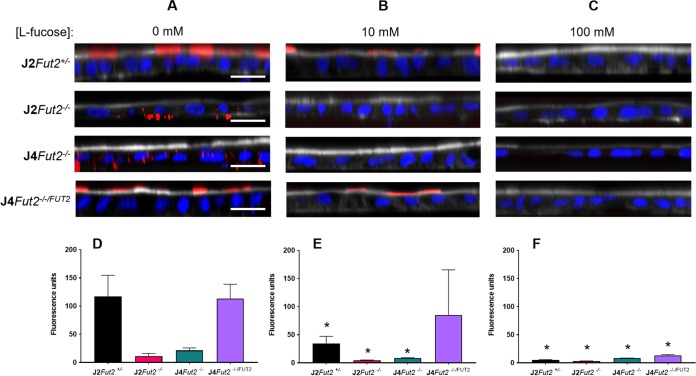
A functional copy of *FUT2* is needed for Fucα1,2Gal antigen expression on the apical surface (J2Fut2^+/−^ and J4Fut2^−/−/FUT2^ images in panel A). Fucα1,2Gal antigen expression was not detected on the apical surfaces of cell lines with no functional FUT2 gene (J2Fut2^−/−^ and J4Fut2^−/−^ images in panel A). H antigen expression was analyzed by UEA-1 lectin (red) in HIE lines. (B and C) Specificity for UEA-1 lectin detection of Fucα1,2Gal antigen in all enteroid lines is demonstrated by the reduction of staining when UEA-1 was preincubated with either 10 mM (B) or 100 mM (C) l-fucose. (D to F) Graphical quantitation of fluorescence is shown below the image panels. Each data bar represents the mean fluorescence from six total wells collected from two experiments. Error bars denote SD. For each enteroid line, significant differences in fluorescence comparing no l-fucose pretreatment of UEA-1 (D) to 10 mM (E) or 100 mM (F) pretreatment were determined by Student’s *t* test (*, *P* < 0.05). In all image panels, the nuclei are marked with DAPI (blue), Fucα1,2Gal antigen was detected by UEA-1 lectin (red), and the brush border is indicated by actin expression using phalloidin (white). Bars, 20 μm.

We next used immunofluorescence microscopy with fluorescently labeled Ulex europaeus agglutinin-1 (UEA-1 lectin) to detect polarized cell surface expression of terminal α1,2-fucose (Fig. 2). Both J2*Fut2^+/−^* and J4*Fut2^−/−/FUT2^* cells had apical staining of UEA-1 lectin. This apical staining was lost in J2*Fut2^−/−^* and J4*Fut2^−/−^* cells (Fig. 2A). These findings indicate that, as expected, terminal fucosylation of the glycan precursor in HIEs depends on the expression of the *FUT2* gene. In the secretor-negative lines, unexpected internal cellular UEA-1 staining was present that was not observed in the secretor-positive lines. To confirm that the internal staining was due to specific UEA-1 recognition of α1,2-fucose and not off-target binding due to loss of the specific ligand, we preincubated the UEA-1 lectin with l-fucose prior to staining. When UEA-1 was preincubated with 10 mM l-fucose, staining was not detected in the secretor-negative lines and was significantly reduced in the secretor-positive lines (Fig. 2B). After preincubation with 100 mM l-fucose, UEA-1 staining was not detected in either the secretor-negative or secretor-positive lines (Fig. 2C). Together, these data indicate that the apical and the internal staining observed with UEA-1 is due to specific interaction with α1,2-fucose. Table 1 summarizes the genotypic and phenotypic findings for the parental, KO and KI cell lines.

### Effect of FUT2 expression on replication of HuNoV strains in isogenic HIE cell lines.

To assess whether these isogenic cell lines support HuNoV replication, we evaluated virus binding and subsequent replication of GII.4, GII.3, GII.17, and GI.1 HuNoV strains that we previously demonstrated replicate in HIEs (2). Since all of these viruses but GII.4 require bile for replication, all infections were performed in the presence of glycochenodeoxycholic acid (GCDCA), a bile acid that supports replication (21).

**FIG 3:**
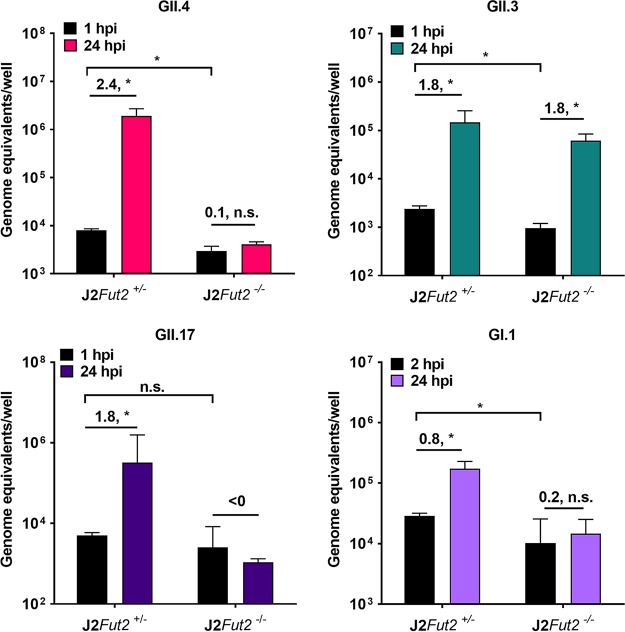
Knocking out *FUT2* prevents infection of GII.4, GII.17, and GI.1 HuNoV strains in J2 HIEs. HIE monolayers were inoculated with GII.4, GII.3, GII.17, or GI.1 HuNoV stool filtrate in 500 μM GCDCA-containing Intesticult medium for 1 h (GII.4, GII.3, and GII.17) or 2 h (GI.1) at 37°C. After two washes with CMGF(−) medium, the cells were cultured in the presence of GCDCA for 24 h at 37°C. Total well RNA was extracted, and genome equivalents (GEs) were determined by RT-qPCR. Each data bar represents the mean for three wells of inoculated HIE monolayers. Error bars denote SD. Each experiment was performed two or more times, with three technical replicates in each experiment. Data from a representative experiment are shown in this figure. Numbers above the bars indicate log_10_ fold change comparing GEs at 24 h postinfection (hpi) to 1 or 2 hpi. Significance was determined by two-way ANOVA with *post hoc* analysis using Tukey’s test (*, *P* < 0.05; n.s., not significant).

First, we assessed the effect of KO of *FUT2* from J2 cells with the four HuNoV strains (Fig. 3). After 1 or 2 h postinfection (hpi) and washing off the inoculum, GII.4, GII.3, and GI.1 binding was significantly reduced in the J2*Fut^−/−^* cells compared to the parental J2*Fut2^+/−^* cells. GII.17 binding was slightly, but not significantly, reduced in several experiments. Viral replication was completely abrogated at 24 hpi for GII.4, GII.17, and GI.1 in the J2*Fut2^−/−^* HIEs. GII.3 was able to replicate in the J2*Fut2^−/−^* line, consistent with our previous findings that GII.3 is able to replicate in some secretor-negative lines (2). These findings support the requirement for functional FUT2 and secretor-positive HBGAs as an initial binding factor for several strains of HuNoV that is critical for replication.

**FIG 4:**
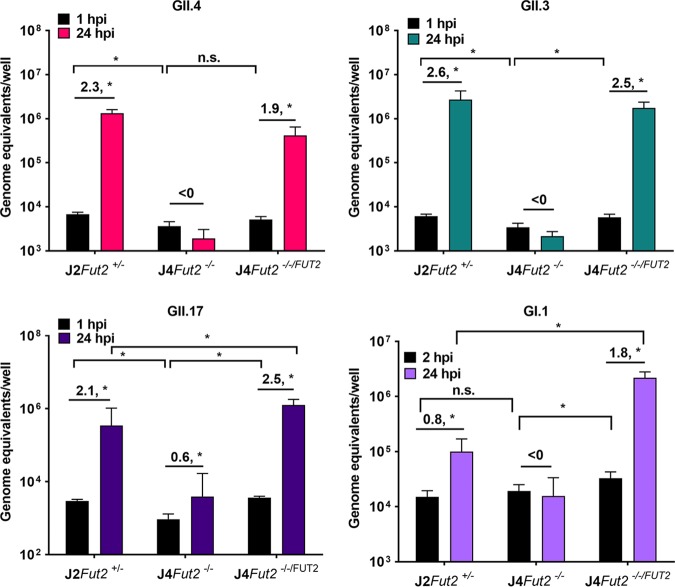
Knocking in *FUT2* is sufficient for infection of J4 HIEs with all HuNoV strains tested. HIE monolayers were inoculated with GII.4, GII.3, GII.17, or GI.1 as described in the legend to Fig. 3. Total well RNA was extracted, and GEs were determined by RT-qPCR. Each data bar represents the mean for three wells of inoculated HIE monolayers. Error bars denote SD. Each experiment was performed two or more times, with three technical replicates in each experiment. Numbers above the bars indicate log_10_ fold change comparing GEs at 24 hpi to 1 or 2 hpi. Significance was determined by two-way ANOVA with *post hoc* analysis using Tukey’s test (*, *P* < 0.05; n.s., not significant).

Next, we assayed the J4*Fut2^−/−/FUT2^* line to determine whether a genetically resistant HIE line can become susceptible with the expression of FUT2. We observed increased HuNoV binding at 1 or 2 hpi in the J4*Fut2^−/−/FUT2^* line compared with the parental nonsecretor J4*Fut2^−/−^* line for all four HuNoV strains (Fig. 4); however, for GII.4, the increase was not significant. J4*Fut2^−/−/FUT2^* was permissive to infection by all four viruses, based upon increases in viral genome equivalents (GEs) at 24 hpi. Interestingly, although GE levels at 1 to 2 hpi were similar, we observed greater increases in GEs for GII.17 and GI.1 HuNoVs in J4*Fut2^−/−/FUT2^* cells than in J2*Fut2^+/−^* cells at 24 hpi. This suggests that additional factors that vary between individual HIE lines, aside from secretor status, may influence the replication of these two strains. Taken together, these results show that functional FUT2 is sufficient and critical for replication of multiple HuNoV strains and that GII.3 is capable of infection in some secretor-negative HIE lines.

## DISCUSSION

Previous studies demonstrated that the HuNoV genome is capable of productive infection in transformed cell lines (e.g., Huh-7) following transfection. Overexpression of the *FUT2* gene increased virus binding to the cells but did not make them susceptible to infection (19). These data indicated the presence of a block between virus binding and entry. The development of the HIE HuNoV infection model identified that enterocytes in these cultures are infected with HuNoVs and allowed the performance of studies to evaluate factors important for virus infection (2). The current study demonstrates that FUT2 alone is necessary and sufficient for the infection and replication of the secretor-dependent GI.1, GII.4, and GII.17 HuNoVs in HIE cells.

Virus attachment and entry into the infected cell are complex processes, and the exact role of fucosylated molecules in HuNoV entry remains to be determined. It is likely that initial binding of secretor-dependent HuNoVs to HIEs is regulated by fucosylation, with the attachment factors being fucosylated. However, it is unclear whether the fucosylated molecules serve only as the initial attachment factor that then facilitates interaction with a virus-specific receptor or whether the fucosylated molecules function as a virus receptor themselves; examples of both mechanisms have been described previously with nonfucosylated glycans (22). For example, reovirus binding to its proteinaceous JAM-A receptor is enhanced by initial binding to sialic acid residues (23), while human coronaviruses OC43 and HKU1 bind to 9-O-acetylated sialic acid receptors (24) and influenza viruses bind to terminal sialic acid receptors (25). If fucosylated molecules serve only as attachment factors, then the virus receptor(s) for secretor-dependent HuNoVs is present in both secretor-positive and secretor-negative HIEs and is not expressed in other nonsusceptible, secretor-positive cancer-derived or transformed cell lines. On the other hand, if the fucosylated molecules function as the virus receptor, it is unclear why secretor-positive cultured transformed cells derived from cancer patients are not susceptible to infection. It is possible that transformed lines do not properly express the needed receptors or glycans or other signaling pathways essential for infection. Transformation may abrogate proper glycosylation in nonsusceptible cell lines compared to nontransformed HIE lines (26).

We observed a striking phenotypic difference in FUT2-expressing HIE cells compared to cells not expressing FUT2. UEA-1 detection of α1,2-fucose was observed exclusively at the apical surface in FUT2-positive cells. Unexpectedly, in the secretor-negative lines, there was internal cellular staining by UEA-1. The staining was due to UEA-1-specific interactions with α1,2-fucose, indicating that these glycan structures are present but unable to transit to the surfaces of the secretor-negative HIE cultures. There is evidence that FUT2 expression leads to surface expression of α1,2-fucosylated molecules. This was shown in specific-pathogen-free mice, where the lumen of the small intestine is mostly lacking in surface fucosylation, and intraperitoneal injection of lipopolysaccharide (LPS) stimulates expression of α1,2-fucosylated molecules at the surface in a FUT2-dependent manner (27, 28). There may be additional fucosyltransferase enzymes present in our HIEs capable of adding fucose to glycoproteins or glycolipids in the absence of FUT2 activity. However, these alternatively fucosylated molecules are unable to transit to the cell surface. Fucosyltransferase 1 (FUT1), expressed in erythroid cells and some other tissues, is also capable of adding α1,2-fucose, although preferentially on glycan chains other than those found in intestinal epithelia. In the parental J2 enteroid line used in this study, *FUT1* transcripts are expressed by transcriptome sequencing (RNA-seq), and the fragment per kilobase per million (FPKM) value is ∼60-fold lower than that of *FUT2*. In bovine coronary venular endothelial cells, a lotus lectin (LTL) that also recognizes α1,2-fucosylated molecules detects cytoplasmic tubule structures, and this staining is lost after depletion of both FUT1 and FUT2 (29). Further studies showed that LTL costained with a Golgi marker in newly derived primary human fibroblasts from oral mucosa, but this Golgi colocalization was lost and LTL was instead detected in tubule structures as the fibroblasts were passaged. In our system, the presence of some FUT1 expression may allow for α1,2-fucose-containing structures intracellularly. Future studies will determine the identity and subcellular location of the fucosylated molecules present in the FUT2-negative HIE lines and whether FUT1 is required for their presence.

HIEs provide an excellent tool for future studies on intestinal enzymes involved in glycosylation and how glycosylation alters glycoprotein localization. An association of enteric commensals and pathogens with host secretor status has led to increased recognition of secretor glycans being susceptibility factors important in infection and disease outcomes (30, 31). The exact role played by the glycans in these infections is not fully understood. Our isogenic, physiologically active HIE lines should be helpful to determine whether fucosylation plays a role in microbe binding, entry, or postentry processes that may affect the epithelial responses to infection, and it will be interesting to understand different outcomes of infection with the different microbes.

## MATERIALS AND METHODS

### Plasmid constructs.

The cDNA of *FUT2*, obtained from J2 human intestinal enteroid (HIE) total RNA, was amplified with specific gene primers (Table 2) and cloned into the lentiviral expression vector pLVSIN-IRES-puromycin (32) using In-Fusion cloning kit (TaKaRa-Clontech) according to the manufacturer’s instructions. A Cas9-expressing lentiviral vector (lentiCas9-BLAST; plasmid no. 52962) and single guide RNA (sgRNA) expression vector (lentiGuide-puro; plasmid no. 52963) were purchased from Addgene. The sgRNA sequence targeting the human *FUT2* gene (5′-CCAGCCAGCTCAGGGGGATG-3′) was cloned into the lentiGuide-puro vector following the manufacturer’s protocol.

**TABLE 2:** Primers used in this study

Gene	Primer	Template strand	Sequence (5′→3′)	Reference
*FUT2*	FUT2-280F	+	AGCCTCAACATCAAAGGCACTGGGA	Saxena et al. (35)
	FUT2-564R	−	AACCAGTCCAGGGCCTGCTGTA	Saxena et al. (35)
	FUT2-97F	+	ATGGCCCACTTCATCCTC	This study
	FUT2-1095R	−	TTAGTGCTTGAGTAAGGGGGAC	Ito et al. (36)
	BO-119F	+	GGCTAGCGAAGATTCAAG	This study
	BO-120R	−	TCGTTCAGGTGGTAGTTC	This study
*FUT3*	FUT3-260F	+	GTGCAGCCAAGCCACAATG	This study
	FUT3-888R	−	CTGCAGGCTCTGGTAGTAGC	This study
	FUT3-840F	+	CAACTGGAAGCCGGACTCA	This study
	FUT3-1485R	−	CAGGCAAGTCTTCTGGAGGG	This study
*ABO* exon 6	ABO-4522F	+	CAGAAGCTGAGTGGAGTTTCC	This study
	ABO-6R	−	CTCGTTGAGGATGTCGATGTTG	Muro et al. (37)
*ABO* exon 7	ABO-6037F	+	TTCCTCAGCGAGGTGGATTA	This study
	ABO-6378R	−	AGCACCTTGGTGGGTTTG	This study

### Lentivirus packaging and production.

A third-generation lentivirus carrying the *FUT2* gene (Lv-FUT2) was produced by cotransfecting HEK293T cells with a combination of a lentivirus plasmid (pLVSIN-FUT2-IRES-puro, lentiCas9-BLAST, or lentiGuide puro) and three packaging plasmids (pMDLg/pRRE [plasmid no. 12251; Addgene], envelope plasmid pMD2.G [plasmid no. 12259; Addgene], and pRSV-Rev [plasmid no. 12253; Addgene]) at a ratio of 3.5:2:1:1, respectively, using polyethylenimine HCl Max molecular weight (MW) 40,000 (Polysciences) (32). The culture supernatant was harvested 60 to 72 h posttransfection, passed through a 0.45-μm filter, concentrated by using LentiX-concentrator (TaKaRa-Clontech) according to the manufacturer’s protocol, and suspended in CMGF(+) medium for transduction.

### Lentiviral transduction of HIEs.

A cell suspension was prepared from three-dimensional (3D) undifferentiated jejunal HIEs cultivated as previously described (2, 33). After trypsinization and pelleting of the cells at 300 × *g*, the resulting cell pellet was suspended at a concentration of 3 × 10^5^ cells per ml of concentrated lentivirus supplemented with 10 μM Rho-associated protein kinase (ROCK) inhibitor Y-27632 (catalog no. Y0503; Sigma) and 8 μg/ml Polybrene (catalog no. TR-1003-G; EMD Millipore). The mixture was plated in one well of a 48-well plate. The plate was then centrifuged for 1 h at 300 × *g* at room temperature (RT). After spinoculation, the lentivirus solution was removed, the cells were washed once with CMGF(−) medium, centrifuged again, embedded in 30-μl Matrigel plug, and incubated at 37°C and 5% CO_2_ in the presence of CMGF(+) medium with ROCK inhibitor for recovery. Five days postransduction, the cells were treated with puromycin (2 μg/ml) or Blasticidin S (5 μg/ml) until mock-treated cells were completely dead. Single cells were isolated by serial dilution in 96-well plates for sgRNA-transduced HIEs, and deletion of the gene was confirmed by sequencing of genomic DNA from each single cell clone using primers BO-119 and BO-120 (Table 2) that amplified the portion of the *FUT2* gene targeted by the sgRNA.

### HIE HBGA phenotyping.

Differentiated HIE cells were suspended in phosphate-buffered saline (PBS) and boiled for 5 min. After incubation of the boiled supernatants on vinyl, flat-bottomed, 96-well plates (ThermoFisher Scientific) for 4 h at RT and blocking with 10% Carnation instant nonfat dry milk overnight at 4°C, anti-Le^a^ Gamma-clone (Immucor), anti-Le^b^ (BG-6; Biolegend), anti-A type Gamma-clone (Immucor), or anti-B type Gamma-clone (Immucor) was used as the primary antibody, and horseradish peroxidase (HRP)-conjugated goat anti-mouse IgG (Sigma) was used as the secondary antibody. 3,3′,5,5′-Tetramethylbenzidine (TMB) (KPL) was used as a HRP substrate, and the reaction was stopped with 1 M phosphoric acid (Fisher Scientific) after a 10-min incubation at RT. The absorbance was determined at 450 nm with a Spectramax 190 plate reader (Molecular Devices). Pooled saliva samples from persons who collectively express each histo-blood group antigen (HBGA) type evaluated was used as a positive control (34). Saliva from an individual negative for the HBGAs was used as a negative control.

### HIE HBGA genotyping.

DNA was extracted from HIEs and primers (Table 2) targeting exons 6 and 7 of the *ABO* gene, the *FUT2* (secretor) gene, and the *FUT3* (Lewis) gene were used to generate amplicons that were purified using the GeneJET PCR purification kit (ThermoFisher Scientific) and sequenced (GeneWiz). Chromatograms were examined to identify single nucleotide polymorphisms associated with loss of function (for *FUT2*. A385T, G428A, C571T, and C628T; for *FUT3*, T59G, T202C, C314T, G484A, G508A, G667A, G808A, and T1067A or A, B, or O genotype (for exon 6, nucleotides 261 and 297; for exon 7, nucleotides 657, 703, 771, 796, 803, 829, and 930).

### Immunofluorescence and quantitation.

HIE monolayers were grown in glass bottom plates (catalog no. 655892; Greiner Bio-One), differentiated for 5 days, and fixed with 4% paraformaldehyde (PFA) for 20 min at RT. HIE monolayers were permeabilized and blocked with 5% bovine serum albumin (BSA) in 0.1% Triton X-100 in PBS for 30 min at RT. All the subsequent steps were performed in PBS plus 0.1% Triton X-100. HBGAs and cell boundaries were detected after overnight incubation at 4°C with rhodamine-labeled Ulex europaeus agglutinin-1 (UEA-1) (1:1,000) (catalog no. RL-1062; Vector Laboratories) and Alexa Fluor 647 phalloidin (ThermoFisher Scientific), respectively. For the fucose inhibition assay, the UEA-I was incubated with different concentrations of l-fucose at RT for 1 h prior to staining. Nuclei were stained with 4′,6′-diamidino-2-phenylindole (DAPI) (300 nM) for 5 min at RT. Orthogonal 5-μm-thick sections of the sample were captured using a Zeiss LSM 510 confocal microscope. For quantifying fluorescence intensity, five fields per well were analyzed. The fluorescence threshold in these images was set in Image J. Mean fluorescence data from 15 identical regions of interest (ROIs) per field were collected. Comparisons between treatment groups were made using a Student’s *t* test. *P* values of <0.05 were considered statistically significant.

### *In vitro* HuNoV infection.

Jejunal HIE monolayers in 96-well plates were differentiated for 5 days in commercial Intesticult (INT) human organoid growth medium (Stem Cell Technologies) and inoculated with the indicated positive HuNoV stool filtrates at 37°C for 1 h (GII/Hu/US/2012/GII.4 Sydney [P31]/TCH12-580, 9 × 10^5^ genome equivalents [GEs]/well; GII/Hu/US/2004/GII.3 [P21]/TCH04-577, 4.3 × 10^5^ GEs/well; GII/Hu/US/2014/GII.17 [P38]/TCH14-385, 1.8 × 10^6^ GEs/well) or for 2 h (GI/Hu/US/2006/GI.1 Norwalk [P1]/BCM723-02, 6.9 × 10^5^ GEs/well) as described previously (2). Each infection was performed in triplicate wells for each time point, and conditions were tested in at least two independent experiments. Inocula were removed, and monolayers were washed twice with CMGF(−) medium to remove unbound virus. Differentiation INT medium (100 μl supplemented with 500 μM glycochenodeoxycholic acid [GCDCA] [Sigma]) was then added to each well, and the cultures were incubated at 37°C for the indicated time points. RNA was extracted from each well using the KingFisher Flex Purification system and MAgMAX-96 viral RNA isolation kit. RNA extracted at 1 to 2 hpi was used as a baseline to determine the amount of input virus that remained associated with cells after the infected cultures were washed to remove unbound virus. Replication of virus was determined by HuNoV RNA levels, which were quantified using a standard curve based on a recombinant HuNoV RNA transcript, and replication of virus was determined by assessing changes in virus GE levels at 24 hpi from baseline.

### Statistics.

All statistical analyses were performed on GraphPad Prism (GraphPad Software, La Jolla, California USA). Samples with RNA levels below the limit of detection of the reverse transcription-quantitative PCR (RT-qPCR) assay were assigned a value that is one-half the limit of detection of the assay. Comparisons between infection time point groups or infected cell lines were made using two-way analysis of variance (ANOVA) with Tukey’s test for *post hoc* analyses. *P* values of <0.05 were considered statistically significant.
